# Nasopharyngeal microbiome and resistome profiles in dairy calves fed milk replacer with low-level β-lactams

**DOI:** 10.3168/jdsc.2025-0994

**Published:** 2026-05-15

**Authors:** Grazielle Cioletti, Sophia Kenney, Ernest Hovingh, Hayley Springer, Bradd J. Haley, Erika Ganda

**Affiliations:** 1 Department of Animal Science, Pennsylvania State University, University Park, PA 16802; 2 Department of Veterinary and Biomedical Sciences, Pennsylvania State University, University Park, PA 16802; 3 Environmental Microbial and Food Safety Laboratory, Beltsville Agricultural Research Center, Agricultural Research Service, United States Department of Agriculture, Beltsville, MD 20705; 4 One Health Microbiome Center, Huck Institutes of the Life Sciences, Pennsylvania State University, University Park, PA 16802

## Abstract

Feeding waste milk (WM) to preweaning dairy calves is a common management practice that offers economic benefits but may influence the microbiome and antimicrobial resistance (AMR) due to the presence of antibiotic residues. The objective of this study was to describe longitudinal patterns in the nasopharyngeal microbiome and AMR gene profiles of dairy calves fed either nonsupplemented milk replacer or milk replacer supplemented with low-level β-lactam antibiotics to simulate WM exposure during the preweaning period. Using shotgun metagenomic sequencing, we profiled the nasopharyngeal microbiome and resistome of 11 Holstein bull calves fed milk replacer with (MR+A; n = 6) or without (MR; n = 5) low levels of β-lactam antibiotics. Antibiotic concentrations were selected to reflect residue levels reported in WM. Deep nasopharyngeal swabs were collected every 2 wk from 1 to 15 wk of age; samples from wk 3 and 15 were excluded due to elevated contaminant burden, resulting in 6 retained time points. No significant differences in microbial α-diversity, β-diversity, or community structure were detected by dietary treatment or sampling age. Tetracycline, macrolide-lincosamide-streptogramin, aminoglycoside, metal, acid, and biocide resistance classes were among the most prominent, with descriptive differences in z-score patterns between groups but no significant differences detected. Larger-scale studies are needed to evaluate the long-term effects of WM feeding on respiratory health and AMR dynamics in dairy calves.

## Highlights

• Low-level β-lactam exposure in milk replacer simulated waste milk feeding.
• No significant differences in nasopharyngeal α- or β-diversity by diet or sampling age.
• Resistome profiles similarly showed no significant differences between groups; antibiotic, metal, acid, and biocide resistance gene classes were detected across both groups.
• Larger studies are needed to assess long-term AMR dynamics in dairy calves.

## Graphical Abstract

**Figure:**
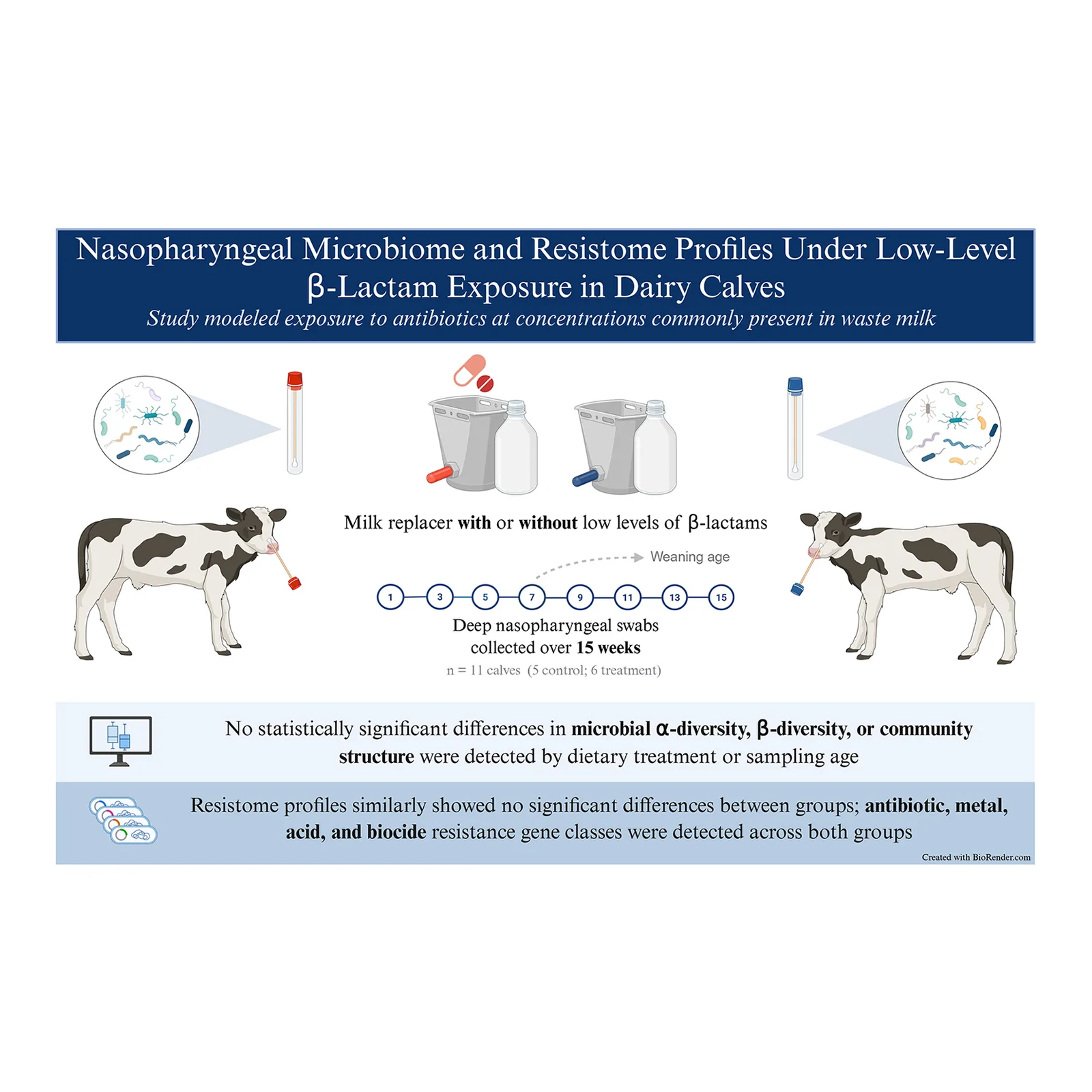
**Summary:** Feeding waste milk to preweaning dairy calves is common practice, but concerns persist about its role in promoting antimicrobial resistance (AMR) due to the presence of antibiotic residues. This exploratory study investigated whether milk replacer supplemented with low levels of β-lactam antibiotics, simulating waste milk exposure, influences the nasopharyngeal microbiome and resistome of dairy calves. Eleven Holstein bull calves were fed milk replacer with (MR+A; n = 6) or without (MR; n = 5) low-level β-lactams. Deep nasopharyngeal swabs were collected every 2 weeks from 1 to 15 weeks of age and analyzed by shotgun metagenomic sequencing. No significant differences in microbial α-diversity, β-diversity, or community structure were detected by dietary treatment or sampling age. Resistome profiles similarly showed no significant differences between groups. These findings provide foundational data to support larger, more powered studies on early-life antimicrobial exposure and respiratory AMR dynamics in dairy calves.

Feeding waste milk (**WM**) to preweaning dairy calves is a common management practice that offers economic and practical benefits compared with milk replacer (Godden et al., 2005). Deemed “waste” because it does not meet industry standards for human consumption, WM may include colostrum, transition milk, or milk from cows with mastitis or under antimicrobial treatment, leading to variable composition and the potential presence of antimicrobial residues (Ulloa et al., 2025). These residues are a public health concern, as they can influence the development and persistence of antimicrobial resistance (**AMR**; BIOHAZ, 2017). Despite these risks, WM remains in use in the United States. In a national survey, 20.9% and 15.0% of heifer calves received unpasteurized and pasteurized WM, respectively (USDA, 2010). More recent data indicate that 40.1% of heifer calves are fed whole or waste milk as their primary liquid diet, compared with 34.8% receiving milk replacer (Urie et al., 2018). Although this estimate does not distinguish WM from saleable whole milk, it highlights how WM feeding remains a prevalent but incompletely characterized practice.

Even at concentrations below the minimum inhibitory concentrations, antibiotic residues can select for resistant organisms and favor the spread of resistance determinants (Gullberg et al., 2011; Tempini et al., 2018). β-Lactams are of particular interest because of their routine therapeutic use in dairy operations (USDA, 2014), as well as their critical importance for human medicine (WHO, 2024). Antimicrobial residues in WM have been reported at low concentrations, typically in the nanogram-per-milliliter range, with β-lactam residues detected at levels below established maximum residue limits but sufficient to exert selective pressure on microbial communities (Pereira et al., 2014; Tempini et al., 2018). Although most previous work has evaluated the fecal resistome of calves fed milk containing antimicrobials (Langford et al., 2003; Thames et al., 2012), very little is known about how low-level β-lactam exposure may influence the microbiome and resistome of the upper respiratory tract (Maynou et al., 2019).

Furthermore, most studies investigating AMR and respiratory microbiota have been conducted in beef cattle or based on diagnostic submissions (Timsit et al., 2017; Magstadt et al., 2018), limiting their applicability to the dairy industry, where respiratory disease remains a major cause of morbidity in preweaning calves (Urie et al., 2018). Understanding how low-level antimicrobial exposure affects the nasopharyngeal microbiome maturation is essential to understanding AMR dissemination and microbial community composition in dairy calves. To date, there is a scarcity of data on longitudinal characterization of the calf nasopharyngeal resistome under simulated WM exposure (Maynou et al., 2019).

The objective of this study was to describe longitudinal patterns in the nasopharyngeal microbiome and AMR gene profiles of dairy calves fed either nonsupplemented milk replacer (control) or milk replacer supplemented with low-level β-lactam antibiotics to simulate WM exposure (treatment group) during the preweaning period.

Singleton bull Holstein calves (n = 11; birth weight: 39.9–51.7 kg) were collected twice daily within 12 h of birth from a single commercial dairy as they became available over the enrollment period. Calves were enrolled if they had not received any antimicrobial treatment and showed no clinical signs of respiratory or systemic disease, as assessed using a standardized scoring system (McGuirk and Peek, 2014). Only calves that remained healthy and antimicrobial-free throughout the study were included in sequencing analyses. Upon arrival, each calf received 2.84 L of commercial colostrum replacer (Calf's Choice Total HiCal, Saskatoon Colostrum Company), followed by a second feeding 6 to 12 h later. Colostrum replacer was used to prevent exposure to antibiotic residues that may be present in natural colostrum from dry cow therapy. Colostrum replacer was used to avoid exposure to antimicrobial residues that may be present in maternal colostrum and was administered uniformly across treatment groups. Passive transfer was assessed in 10 of 11 calves on d 2 of life using serum total protein measured by refractometry (range: 4.4–6.1 g/dL).

Calves were housed individually in solid-sided pens with sealed edges, and feeding vessels (i.e., bottles and buckets) and associated equipment were designed to prevent cross-contamination. Calves were monitored twice daily for clinical signs of respiratory and systemic disease using a standardized scoring system (McGuirk and Peek, 2014), and no clinical signs requiring intervention were observed throughout the study period.

Calves were systematically randomly assigned to 1 of 2 treatment groups: milk replacer without antibiotics (**MR**; n = 5) or milk replacer supplemented with low-level β-lactams (**MR+A**; n = 6). The first-born calf was assigned to the MR+A group, the next to MR, and so forth. Both groups received the same milk replacer formulation (22% CP, 17% fat; mixed at 130 g/L). Feeding volumes increased with age: 2 L twice daily until 10 d, 3 L twice daily from 11 to 21 d, and 4 L twice daily thereafter. During the final week before weaning (7 wk of age), milk replacer was reduced to 4 L once daily.

Separate stock solutions of ampicillin sodium (WG Critical Care), ceftiofur (Zoetis), and penicillin G sodium (VWR Life Sciences) were prepared at concentrations of 1.5 mg/mL, 1.5 mg/mL, and 0.1 mg/mL, respectively, and were frozen at −20°C in small aliquots until use. For the MR+A group, stock solutions of ampicillin sodium, ceftiofur, and penicillin G sodium were added to milk replacer before feeding, to achieve final concentrations of 150 ng/mL ampicillin, 150 ng/mL ceftiofur, and 10 ng/mL penicillin G. These concentrations reflect levels commonly detected in WM (Pereira et al., 2014; Tempini et al., 2018).

Although these antimicrobials are administered therapeutically to cattle via parenteral routes at the labeled dose, generally on the order of milligrams per kilogram of BW (FDA, 2026a,b,c), the exposure levels used in this study were substantially lower (∼100–1,000-fold below therapeutic ranges) and were intended to model repeated low-dose oral ingestion associated with consumption of WM containing antimicrobial residues.

Deep nasopharyngeal swabs were collected using a 30-cm double-guarded equine uterine culture swab to minimize environmental contamination. External nares were dry wiped free of gross organic matter before insertion. Sampling began at 7 d of age and continued every 2 wk until 15 wk of age (±2 d), corresponding to sampling wk 1 through 15 (total n = 88 samples). Blinding of personnel to treatment allocation was not feasible due to preparation and administration of treatment-specific milk replacers. Antimicrobial exposure in the MR+A group began following completion of the colostrum-feeding period when calves were transitioned to milk replacer containing antimicrobial residues; therefore, calves had been exposed to treated milk for several days before the first sampling time point. Consequently, microbiome composition before antimicrobial exposure was not characterized, and the MR group served as a concurrent untreated comparison rather than a true pre-exposure baseline. Swabs were transported on ice, stored at −80°C, and later processed for metagenomic analysis.

Samples were pre-processed following (Gaeta et al., 2020) and extracted using the MagMAX Microbiome Nucleic Acid Purification Kit (Thermo Fisher Scientific). DNA concentration was quantified by Qubit fluorometry. Sequencing libraries were prepared with the sparQ DNA Library Prep Kit (Quantabio) using 10 ng of input DNA and unique dual indices, and then sequenced on an Illumina NovaSeq 6000 platform (2 × 150 bp) at the Hershey College of Medicine Genomics Core. Extraction negative controls consisted of sterile nuclease-free water processed alongside samples through the full extraction workflow, including transfer into sample tubes and passage through extraction columns. A commercially available mock microbial community (Zymo Research, cat. no. D6300) was included as a positive control. Raw sequences were deposited in the NCBI Sequence Read Archive (BioProject ID PRJNA1347079), and processed data are available at https://github.com/gandalab/AMC-Calf_Nasopharyngeal.

Raw reads were quality-checked using FastQC (Andrews, 2010) and summarized with MultiQC (Ewels et al., 2016). Adapter sequences were removed with Trimmomatic v0.39 (Bolger et al., 2014) in paired-end mode using the TruSeq3-PE adapter file and default Illumina clipping parameters (seed mismatches = 2, palindrome = 30, simple clip = 7).

Antimicrobial resistance gene profiling was performed using the AMR++ v2.0 pipeline (Doster et al., 2020) with threshold parameter set to 0 and maximum 20 computational threads. Within the AMR++ pipeline, reads underwent quality control, host read removal, and annotation against the MEGARes v2 database. Host reads were removed by aligning against the *Bos taurus* Holstein–Friesian reference genome (isolate LICNZHF1; WGS assembly CM038079.1), using BWA-MEM (Li and Durbin, 2009), followed by Samtools filtering (Li et al., 2009; mapping quality score [MAPQ] ≥30).

Microbial taxonomy was assigned using Kraken2 v2.1.3 (Wood et al., 2019), with the Standard database (built July 2023) using paired-end classification, minimum-hit-groups of 3, and confidence threshold set to the default value (0.0). Abundance estimates were refined with Bracken v2.8 (Lu et al., 2017) using a read length of 151 bp and default k-mer distribution parameters (k = 35). KrakenTools (Lu et al., 2022) was used only for formatting and combining Kraken2/Bracken reports. No a priori power analysis was performed, as the samples were originally collected for a previous doctoral thesis (Springer, 2022) and this was a secondary analysis.

All analyses were performed in R version 4.3.2 using RStudio (R Core Team, 2023). Contaminants were identified using the prevalence-based method implemented in the decontam package (Davis et al., 2018), with prevalence thresholds of 0.5 for AMR++ profiles and 0.8 for taxonomic profiles derived from Kraken2. Samples were additionally filtered based on sequencing depth. Taxa were retained for downstream analysis if present in at least 1% of samples and with total relative abundance ≥0.0001.

Contaminant burden was assessed as the proportion of relative abundance attributed to features identified as contaminants using the prevalence-based method. Weeks 3 and 15, with the highest contaminant burden (mean contaminant fraction >0.60), were excluded from downstream analyses to reduce potential bias associated with low-biomass contamination. Following filtering, 53 samples were retained for taxonomic analyses and 50 for AMR analyses.

Taxonomic and AMR gene (**ARG**) α-diversity, including Shannon index and observed richness, were calculated using the phyloseq package (McMurdie and Holmes, 2013). Potential outliers were identified separately for each metric as values exceeding 3 SD from the mean and were excluded from α-diversity analyses only. Longitudinal effects of dietary treatment, sampling age (week), and their interaction were evaluated using mixed-effects models to account for repeated measurements within calves. Shannon diversity was analyzed using a linear mixed-effects model, and observed richness was analyzed using a generalized linear mixed-effects model with a negative binomial distribution. In both models, calf was included as a random intercept. Model-estimated marginal means and their associated SE were obtained using the emmeans package (Lenth and Piaskowski, 2026), and pairwise dietary contrasts at each sampling week were adjusted for multiple testing using the Benjamini–Hochberg procedure.

Post hoc precision estimates were calculated to characterize the magnitude of difference the study was statistically powered to detect. For Shannon diversity, the approximate detectable difference was estimated as twice the model-derived SE of the pairwise diet contrast obtained from the emmeans package. For observed ARG richness, the detectable fold-change was estimated by exponentiating twice the SE of the dietary treatment coefficient from the negative binomial mixed-effects model. Statistical significance was defined at α = 0.05.

β-Diversity was assessed on centered log-ratio (**CLR**)-transformed abundance data using Euclidean distance matrices, equivalent to Aitchison distance for compositional data. Community differences were evaluated at the genus level for taxonomic profiles and at the class level for ARG profiles. Multivariate differences in community composition were tested using PERMANOVA implemented in the vegan package with 10,000 permutations. To account for repeated longitudinal sampling within calves, permutations were constrained within individual animals. Homogeneity of multivariate dispersion between dietary groups was assessed using analysis of multivariate dispersion. Ordination patterns were visualized using principal coordinates analysis with 95% confidence ellipses.

Genus-level taxonomic composition was summarized by aggregating sequence features at the genus rank and transforming counts to relative abundance for each sample. Dominant genera were defined based on global mean relative abundance across all samples. Temporal compositional patterns were visualized by calculating the mean genus-level relative abundance within each dietary treatment group at each sampling week. The ARG composition was summarized at the resistance class level following transformation to relative abundance.

Features were aggregated at the AMR class level following compositional transformation. To facilitate comparison of relative abundance patterns across samples, standardized abundance (z-score) profiles were calculated for each AMR class across all samples by centering and scaling class-level abundances. For visualization, dominant resistance classes within broader resistance categories were identified based on global mean abundance, and the top classes per category were displayed using heatmaps with hierarchical clustering and metadata annotations for dietary treatment and sampling week.

Shotgun metagenomic sequencing of 88 nasopharyngeal swab libraries generated a total of 2.81 × 10^9^ raw paired-end reads, with a mean of 3.19 × 10^7^ reads per sample (median 2.16 × 10^7^; range 3.30 × 10^2^ to 2.10 × 10^8^). Following decontamination filtering, 1,047 ARG were retained from 1,106 detected features, and 4,397 taxa were retained from 10,652 taxa identified by Kraken2.

After sequencing depth filtering and exclusion of wk 3 and 15 due to elevated contaminant burden identified during contamination diagnostics, 53 taxonomic samples and 50 AMR samples were retained for downstream analyses. Sample numbers per retained time point ranged from 7 to 10 for taxonomic and resistome analyses.

One sample (AMC70; MR; wk 13) was identified as an outlier and excluded from taxonomic α-diversity analyses only. Linear mixed-effects modeling did not detect significant effects of diet, sampling age, or their interaction on Shannon diversity (diet: *F*_1,10.6_ = 0.69, *P* = 0.43; sampling age: *F*_5,43.0_ = 0.25, *P* = 0.94; diet × sampling age: *F*_5,43.0_ = 1.05, *P* = 0.40; Figure 1A). Similarly, generalized linear mixed modeling showed no significant effects on observed richness by diet ( $\chi_{1}^{2}$ = 0.02, *P* = 0.88), sampling age ( $\chi_{5}^{2}$ = 5.73, *P* = 0.33), or their interaction ( $\chi_{5}^{2}$ = 6.38, *P* = 0.27; Figure 1B). Post hoc precision estimates indicated that the study was powered to detect a ∼0.72-unit difference in Shannon diversity (SE = 0.36) and a ∼2.30-fold difference in observed richness (SE = 0.41) between dietary treatments.

**Figure 1:**
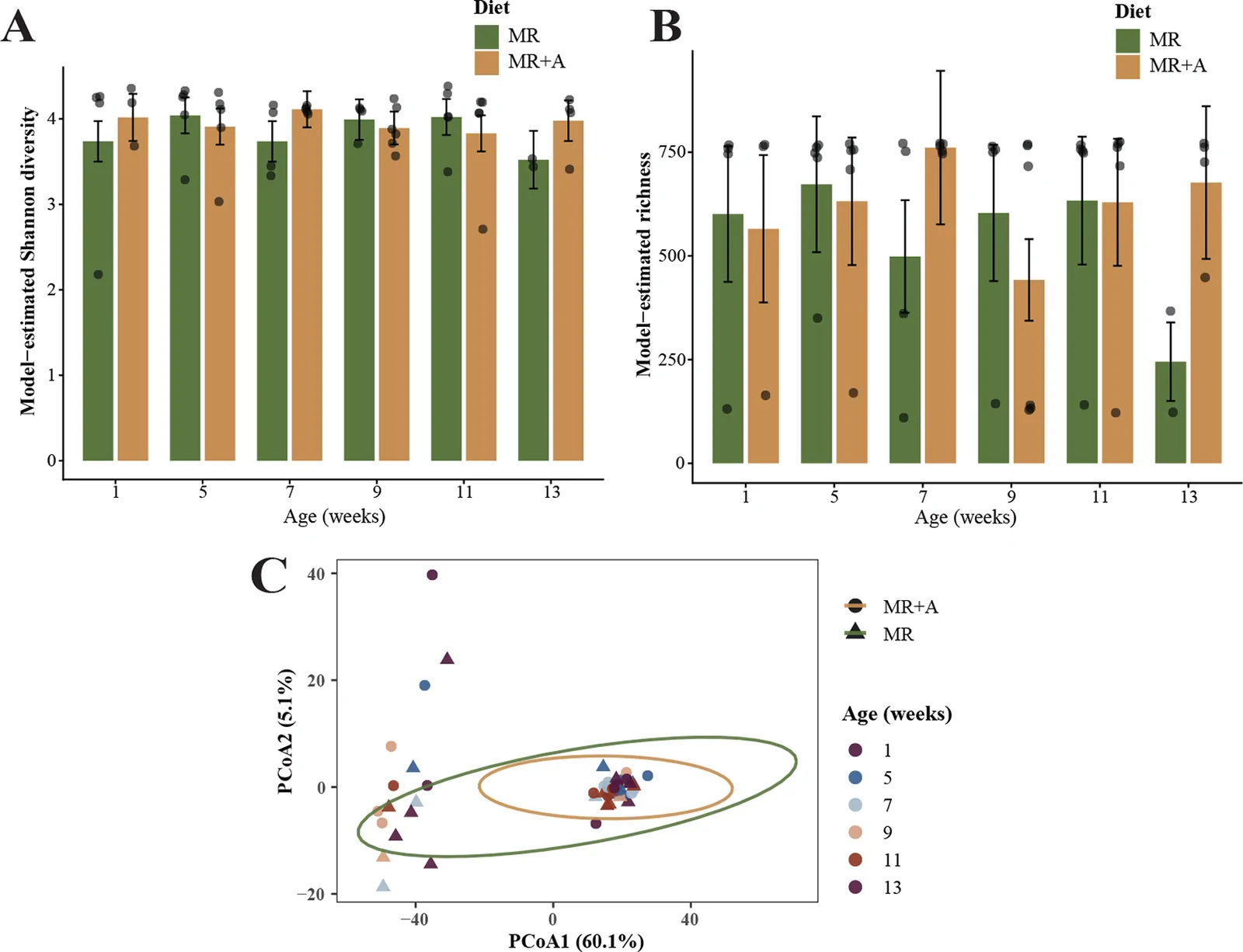
Nasopharyngeal microbiome diversity in dairy calves fed milk replacer (MR; n = 5) or milk replacer supplemented with low-level β-lactams (MR+A; n = 6) from 1 to 13 wk of age. (A) Shannon diversity and (B) observed richness across sampling age. Bars represent model-estimated marginal means from linear (Shannon) and negative binomial (richness) mixed-effects models with calf as a random effect; error bars indicate SEM. No effects of diet, age, or their interaction were detected (*P* > 0.05). (C) Principal coordinates analysis (PCoA) of Aitchison distances from centered log-ratio (CLR)-transformed genus-level data. PCoA is a dimensionality-reduction method used to visualize differences in overall microbial community composition between samples; points closer together are more compositionally similar. Ellipses indicate 95% confidence regions. Points are colored by age and shaped by diet. Groups overlapped (PERMANOVA: *F*_11,41_ = 1.15, R^2^ = 0.24, *P* = 0.25).

β-Diversity of the taxonomic community was assessed using Aitchison distances to evaluate differences in overall microbial composition across sampling weeks and between dietary treatments. PERMANOVA, accounting for repeated measures by including animal ID as a strata variable, indicated no statistically significant effects of age, diet, or their interaction on community composition (*F*_11,41_ = 1.15, R^2^ = 0.236, *P* = 0.25; Figure 1C). Consistent with this, there was no evidence of differences in dispersion between dietary groups, as assessed by permutational analysis of multivariate dispersions (PERMDISP; *F*_1,51_ = 0.48, *P* = 0.52). Overall, no significant effects of dietary treatment or age on between-group variability of taxonomic profiles were detected.

Several genera previously reported in bovine nasopharyngeal microbiomes were detected, including *Streptococcus* (mean relative abundance [**RA**] 3.46%; prevalence 62.3%), *Mannheimia* (RA 0.84%; prevalence 37.7%), *Klebsiella* (RA 0.51%; prevalence 79.2%), *Acinetobacter* (RA 0.39%; prevalence 62.9%), and *Psychrobacter* (RA 0.86%; prevalence 80.2%; Holman et al., 2017; Amat et al., 2020). Genera commonly associated with mammalian skin and upper respiratory mucosal surfaces were also frequently detected, including *Corynebacterium* (RA 1.43%; prevalence 95.0%) and *Neisseria* (RA 0.28%; prevalence 86.8%; Weyand, 2017; Centeno-Martinez et al., 2022). *Ralstonia* (RA 0.978; prevalence 79.6%) was previously identified in deep nasopharyngeal studies in cattle (Magossi et al., 2024) but is also recognized as a common reagent-associated taxon in low-biomass studies (Laurence et al., 2014).

Additional genera detected included *Atopobium* (RA 0.34%; prevalence 84.9%), *Alistipes* (RA 0.42%; prevalence 79.2%), and *Parafannyhessea* (RA 3.63%; prevalence 96.2%), previously reported in fecal microbiomes (Shi et al., 2025; Yang et al., 2026). Manure-associated genera were additionally detected, including *Luteimonas* (RA 0.33%; prevalence 100%; Romero-Yahuitl et al., 2024). *Alistipes* and *Bifidobacterium* (RA 0.22%; prevalence 74.3%) have also been associated with gastrointestinal hindgut communities (Lin et al., 2023). The top 10 genera, selected based on mean relative abundance across all samples, are shown in Figure 2.

**Figure 2:**
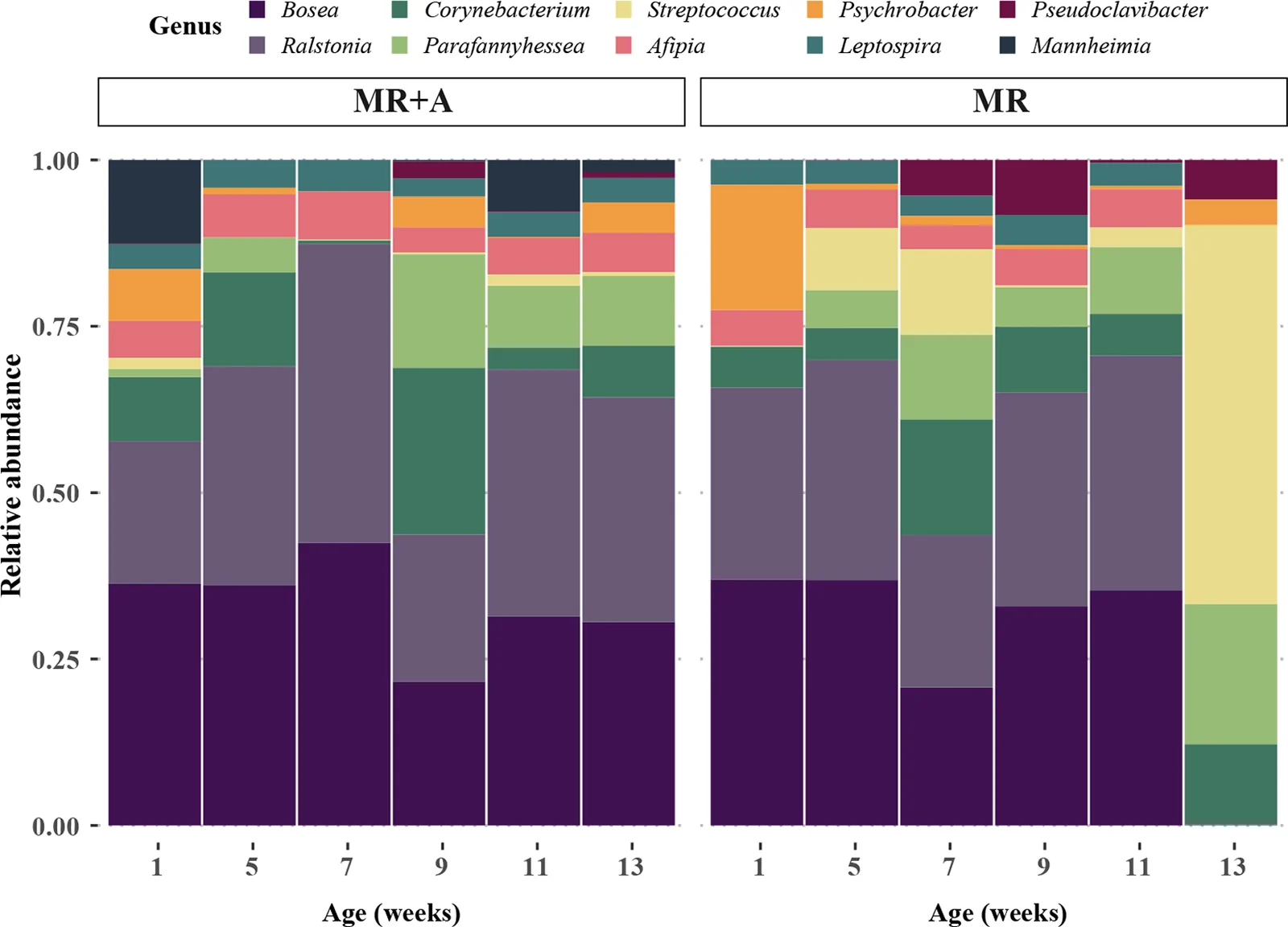
Genus-level community composition of the nasopharyngeal microbiome in calves fed milk replacer (MR) or milk replacer supplemented with low-level β-lactams (MR+A). Stacked bar plots show the mean relative abundance of the 10 most dominant bacterial genera across sampling age. Genus-level composition showed overlap across age and treatment groups, with some week-specific variability (e.g., wk 13 in MR), but no significant differences were detected in multivariable analyses.

Mixed-effects modeling indicated that antimicrobial milk replacer supplementation did not significantly influence within-sample ARG diversity over the study period. The ARG Shannon diversity did not differ by diet (*F*_1,12.3_ = 0.74, *P* = 0.41), and the diet × sampling age interaction was also not significant (*F*_5,43.4_ = 0.52, *P* = 0.76). A nonsignificant trend toward sampling age-related variation in ARG Shannon diversity was observed (*F*_5,43.4_ = 2.16, *P* = 0.07). Similarly, generalized linear mixed modeling showed no effects of diet ( $\chi_{1}^{2}$ = 0.00, *P* = 0.99), sampling age ( $\chi_{5}^{2}$ = 7.76, *P* = 0.17), or their interaction ( $\chi_{5}^{2}$ = 3.38, *P* = 0.64) on observed ARG richness. Pairwise comparisons between diets at individual sampling time points confirmed the absence of significant differences for both ARG Shannon diversity (all adjusted *P* ≥ 0.27) and observed richness (all adjusted *P* ≥ 0.09). α-Diversity indexes can be observed in Figure 3A and B.

**Figure 3:**
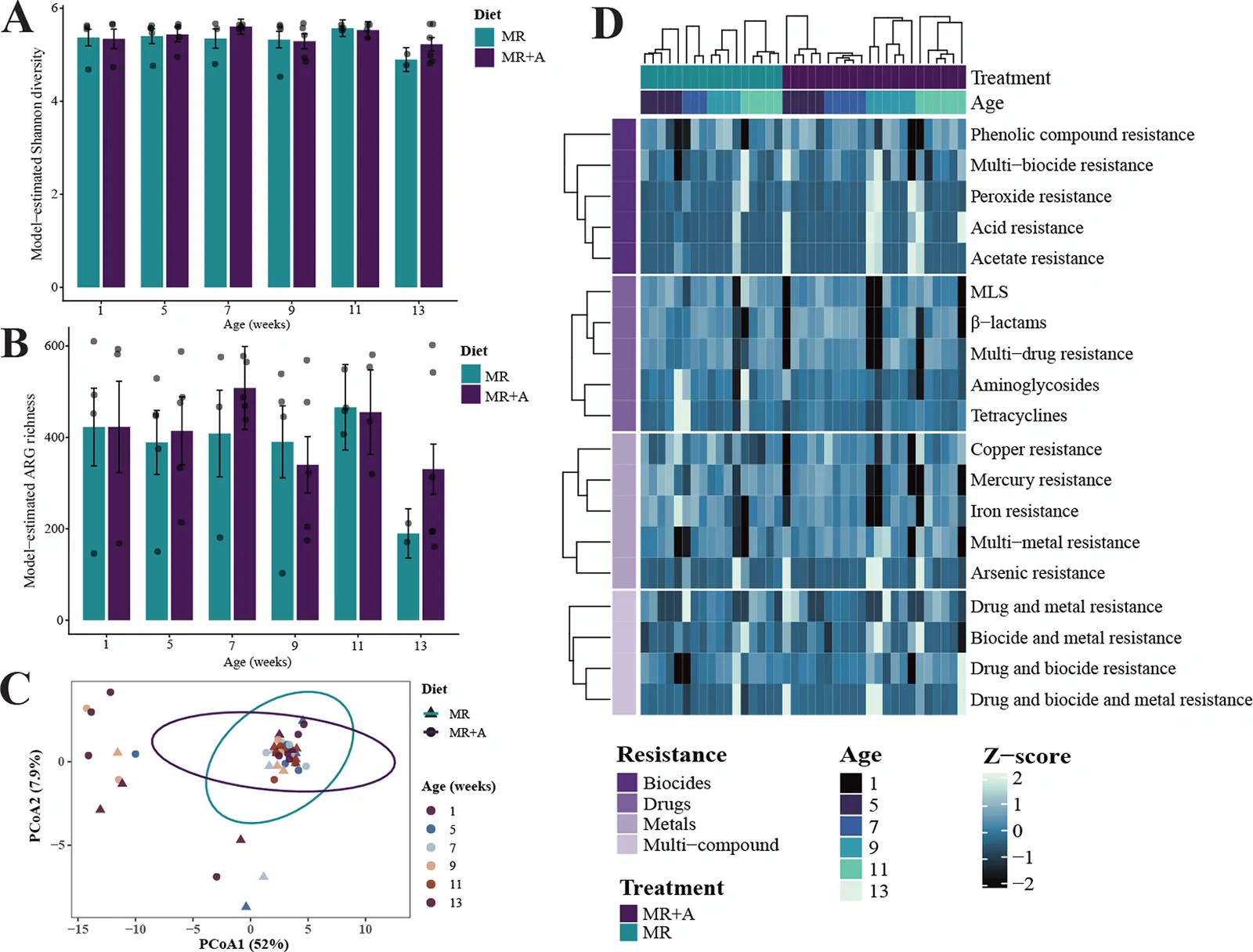
Antimicrobial resistance gene (ARG) diversity and composition in dairy calves fed milk replacer (MR; n = 5) or milk replacer supplemented with low-level β-lactams (MR+A; n = 6). (A) Shannon diversity and (B) observed ARG richness across sampling ages (wk 1, 5, 7, 9, 11, and 13). Bars represent model-estimated marginal means from mixed-effects models with diet, age, and their interaction as fixed effects and calf as a random effect; error bars indicate SEM. No effects of diet, age, or diet × age were detected (*P* > 0.05). (C) Principal coordinates analysis (PCoA) of Aitchison distances from centered log-ratio (CLR)-transformed ARG class-level data. PCoA is a dimensionality-reduction method used to visualize differences in overall ARG community composition between samples; points closer together are more compositionally similar. Ellipses indicate 95% confidence regions. Points are colored by age and shaped by diet. Groups overlapped (PERMANOVA: *F*_11,38_ = 1.04, R^2^ = 0.23, *P* = 0.29). (D) Heatmap of standardized ARG class abundances (z-scores) for the top 5 classes within each resistance category. Z-scores indicate how far each sample's abundance deviates from the mean across all samples; positive values (lighter shades) indicate above-average relative abundance, and negative values (darker shades) indicate below-average relative abundance. Rows are grouped and clustered by category; columns are annotated by diet and age.

Post hoc precision estimates indicated that the study was powered to detect a ∼0.55-unit difference in Shannon diversity between diets (SE = 0.27) and a ∼1.86-fold difference in observed ARG richness (SE = 0.31). Collectively, these results indicate that no significant differences in ARG richness or evenness were detected across early life or between dietary treatments.

Multivariate dispersion did not differ between dietary groups (ANOVA on betadisper: *F*_1,48_ = 0.004, *P* = 0.95; permutation test *P* = 0.95), indicating comparable within-group variability in ARG community structure. Longitudinal PERMANOVA using CLR-transformed Aitchison distances showed no significant effects of calf age or the diet × sampling age interaction on overall ARG community composition (*F*_11,38_ = 1.04, R^2^ = 0.23, *P* = 0.29). Principal coordinates analysis likewise showed substantial overlap in ARG community structure across dietary treatments and sampling time points, consistent with absence of detectable shifts in multivariate composition (Figure 3C).

Z-score values indicated that ARG classes varied in their relative prominence between treatment groups. Genes associated with tetracycline resistance showed the highest mean z-score in MR calves (0.23) compared with MR+A calves (−0.18), followed by macrolide-lincosamide-streptogramin (**MLS**) resistance (MR = 0.17; MR+A = −0.13) and aminoglycoside resistance (MR = 0.17; MR+A = −0.13). Mercury and iron resistance genes also showed higher mean z-scores in MR calves (0.16 and 0.14, respectively) compared with MR+A calves (−0.12 and −0.11). In contrast, multi-metal resistance (MR = −0.45; MR+A = 0.35), acid resistance (MR = −0.19; MR+A = 0.15), drug–biocide–metal resistance (MR = −0.16; MR+A = 0.13), and multi-biocide resistance (MR = −0.16; MR+A = 0.13) showed higher mean z-scores in MR+A calves (Figure 3D).

Similar to previous work demonstrating that tetracycline and MLS resistance genes can be widely distributed within dairy systems and not directly associated with antibiotic exposure (Kyselková et al., 2015; Liu et al., 2019), our findings suggest that resistome patterns may reflect underlying host and environmental factors beyond low-level β-lactam intake under the conditions studied.

Together, these findings indicate that no significant differences in nasopharyngeal microbiome diversity or composition were detected between dietary treatments under the conditions studied, and should not be interpreted as evidence of equivalence given the exploratory nature and limited sample size of this study. Similar to our findings, previous studies reported stable microbial composition over short timeframes in untreated calves (Centeno-Martinez et al., 2023), as well as no significant differences in microbial α-diversity in the nasal microbiota of calves fed pasteurized WM with β-lactam residues and those fed MR (Maynou et al., 2019).

As no respiratory disease events were reported during the study period, the absence of significant differences in community composition may also reflect the low-biomass nature of nasopharyngeal samples and absence of clinical disease. Supporting this, a previous work has shown that upper respiratory microbial load, rather than community composition, was predictive of pneumonia (Lima et al., 2016). Additionally, subclinical lung lesions, which can be detected by thoracic ultrasonography, have been associated with shifts in the nasopharyngeal microbial composition in preweaning calves (Raabis et al., 2021). As subclinical lung lesions were not assessed in the present study, their potential influence on microbiome composition independent of dietary treatment cannot be excluded.

The lack of significant differences in resistome profiles between dietary treatment groups under the conditions studied suggests that low-level dietary β-lactam exposure alone may not be a dominant driver of resistome development in preweaning dairy calves; however, this interpretation should be made cautiously, given the exploratory nature of the study. This highlights the need for ongoing surveillance, particularly as calves may serve as reservoirs of AMR within dairy systems (Salerno et al., 2022).

The relatively small sample size may have limited the ability to detect subtle differences between treatment groups. The lack of significant difference does not indicate equivalence between groups and should be interpreted with caution. In addition, microbiome composition before antimicrobial exposure was not assessed, limiting the ability to evaluate baseline differences between groups.

Calves were enrolled based on availability from a single source herd, which may introduce convenience sampling bias and limit generalizability of findings. Passive transfer status varied among calves based on serum total protein measurements, and was not formally compared between treatment groups. Although passive transfer status could influence early-life microbial colonization and immune function, its specific influence on the nasopharyngeal microbiome under the conditions studied remains unclear, and any differential distribution between groups could represent a potential confounder that cannot be excluded.

Although early-life nutritional intake can influence microbial colonization and immune development, this feeding strategy was applied consistently across treatment groups and is therefore unlikely to have differentially influenced comparisons between diets, although its potential effects cannot be fully excluded. Despite limitations related to sample size and low biomass, the longitudinal design and stringent decontamination controls strengthen the validity of these findings. To our knowledge, there is limited literature on the sampling of calf nasopharyngeal microbiome (Lima et al., 2016; Maynou et al., 2019), and this study provides a longitudinal metagenomic characterization of the nasopharyngeal microbiome and resistome in pre- and postweaning dairy calves fed simulated WM.

This exploratory study characterized the nasopharyngeal microbiome and resistome of dairy calves fed milk replacer containing low-level β-lactams, simulating WM exposure. No significant differences were detected in microbial and ARG diversity over time or between treatment groups under the conditions studied. Despite the absence of significant differences in resistome profiles, the detection of tetracycline, MLS, multi-metal, and biocide resistance genes highlights the need for continued surveillance of AMR determinants in calf populations. Larger-scale studies with expanded sampling, rigorous contamination controls, and improved nasopharyngeal sampling strategies to increase biomass yield are needed to better characterize the long-term effects of early-life antimicrobial exposure on respiratory microbiome and resistome dynamics in dairy calves.
